# Description and Modeling of Relevant Demographic and Laboratory Variables in a Large Oncology Cohort to Generate Virtual Populations

**DOI:** 10.3390/pharmaceutics16121548

**Published:** 2024-12-03

**Authors:** Laura Pérez-Ramos, Laura Ibarra-Gómez, Rubin Lubomirov, María García-Cremades, Eduardo Asín-Prieto, Salvador Fudio, Pablo Zubiaur

**Affiliations:** 1 PharmaMar S.A., Clinical Pharmacology Department, Clinical Development, 28770 Madrid, Spain; 2 Department of Pharmaceutics and Food Technology, School of Pharmacy, Complutense University of Madrid, 28040 Madrid, Spain; 3 Institute of Industrial Pharmacy, Complutense University of Madrid, 28040 Madrid, Spain

**Keywords:** pharmacometrics, cancer, modeling, virtual populations

## Abstract

**Background/Objectives**: Pathophysiological variability in patients with cancer is associated with differences in responses to pharmacotherapy. In this work, we aimed to describe the demographic characteristics and hematological, biochemical, and coagulation variables in a large oncology cohort and to develop, optimize, and provide open access to modeling equations for the estimation of variables potentially relevant in pharmacokinetic modeling. **Methods**: Using data from 1793 patients with cancer, divided into training (*n* = 1259) and validation (*n* = 534) datasets, a modeling network was developed and used to simulate virtual oncology populations. All analyses were conducted in RStudio 4.3.2 Build 494. **Results**: The simulation network based on sex, age, biogeographic origin/ethnicity, and tumor type (fixed or primary factors) was successfully validated, able to predict age, height, weight, alpha-1-acid glycoprotein, albumin, hemoglobin, C-reactive protein and lactate dehydrogenase serum levels, platelet–lymphocyte and neutrophil–lymphocyte ratios, and hematocrit. This network was then successfully extrapolated to simulate the laboratory variables of eight oncology populations (*n* = 1200); only East Asians, Sub-Saharan Africans, Europeans, only males, females, patients with an ECOG performance status equal to 2, and only patients with pancreas cancer or ovarian cancer. **Conclusions**: this network constitutes a valuable tool to predict relevant characteristics/variables of patients with cancer, which may be useful in the evaluation and prediction of pharmacokinetics in virtual oncology populations, as well as for model-based optimization of oncology treatments.

## 1. Introduction

Pathophysiological variability in patients with cancer is associated with differences in response to pharmacotherapy. These differences can be driven by alterations in the pharmacokinetic processes of drug absorption, distribution, metabolism, and excretion. Garantini et al. (2007) [1] extensively discussed the underlying mechanisms of these alterations, which include but are not limited to inflammation (e.g., relationship between serum C-reactive protein [CRP] and alpha-1 acid glycoprotein [AAG] levels with decreased *CYP3A4* expression and reduced docetaxel clearance); vascularity (higher blood flow into the tumor vascular network increasing drug availability, which was observed for doxorubicin and methotrexate, among others); genetic polymorphism (e.g., *UGT1A1* or *TPMT* variation and irinotecan or mercaptopurine metabolism, respectively); drug interactions (e.g., the sharp increase in the docetaxel plasma concentration in patients with breast cancer due to the concomitant use of doxorubicin); host factors affecting drug disposition in tumors (e.g., blood–brain barrier [BBB] permeability); or alterations in the interstitial matrix (i.e., higher interstitial fluid pressure [IFP] inhibiting the transvascular transport of drugs and, consequently, tumor drug uptake, as extensively discussed elsewhere [2]). Additionally, the patterns of drugs binding to plasma proteins, such as AAG and their circulating concentrations, are altered in patients with cancer [3,4,5,6], which impacts drug distribution.

The use of “virtual populations” is gaining popularity in fields such as pharmacology and clinical research, as it allows for the modeling of clinical scenarios in more relevant populations. This approach enhances the ability to predict treatment outcomes and optimize drug development by closely simulating real-world patient characteristics and responses. A virtual population for a specific group of patients is an unobserved, simulated population characterized by a set of distributions for relevant parameters that match those occurring in the real population. For instance, in Teutonico et al.’s (2015) study [7], virtual populations of patients with chronic obstructive pulmonary disease (COPD) were generated using multivariate and discrete re-sampling techniques, where disease severity and demographic characteristics were modeled. This population was then applied in a drug–disease model to describe the treatment effects of a bronchodilator on forced expiratory volume in 1 s (FEV1). Additionally, another study generated virtual populations that captured specific parameters of pediatric patients with obesity for physiologically based pharmacokinetic (PBPK) modeling [8]. However, studies focused on modeling virtual oncology patients remain limited. More specifically, studies that model parameters that may significantly influence the absorption, distribution, metabolism, and excretion (ADME) processes of oncology medications are particularly scarce. A precedent exists in the work of Cheeti et al. (2013) [9], which provides a foundational example in this area.

Cheeti et al. (2013) [9] studied demographic and laboratory variables (e.g., albumin or AAG serum levels) in a cohort of patients with cancer; they modeled equations based on observed data to predict a virtual oncology population that was subsequently integrated into the whole-body PBPK software Simcyp™ (Certara USA and Certara UK Ltd. “Simcyp”). They conducted PBPK simulations of saquinavir and midazolam plasma concentration–time curves in patients with cancer and compared the results with those obtained when the standard Simcyp™ population, comprising healthy adults, was used for modeling.

The pathophysiological variability across different biogeographic groups, commonly referred to as ethnic or racial groups, also determines part of the variability in drug responses [10]. Yet, the scarcity of studies modeling characteristics specific to oncology populations is remarkable, especially among underrepresented ethnic groups. This fact, combined with the rapid advancement in pharmacometric approaches used to support clinical drug development, motivated this work, in which we aimed to (a) analyze the demographic characteristics and the hematological, biochemical, and coagulation variables in a large, diverse oncology cohort; (b) develop and validate a simulation network of laboratory variables, providing open access to the network equations; (c) simulate eight oncology populations with different clinical characteristics using the simulation network, and (d) discuss the relevance and predictive capacity of the modeled clinical and laboratory variables in relation to drug pharmacokinetics, response, and toxicity. Providing the observed data of the modeled population, which includes patients with different biogeographic origins, as well as the equations that allow for obtaining virtual patients, introduces significant novelty with respect to previous studies.

## 2. Materials and Methods

### 2.1. Study Population

Data on 1793 patients with cancer enrolled in 13 clinical trials involving lurbinectedin in monotherapy or in combination were used (54% in phase-III, 28% in phase-II, and 18% in phase-I). All trials were all conducted in adherence with the Declaration of Helsinki, Good Clinical Practice guidelines, and relevant local regulations for clinical trials. Approvals for all study protocols were obtained from the independent local ethics committee (IEC) of each participating center. Prior to any procedure, each patient provided signed, written informed consent for their enrollment in the clinical trial and subsequent research projects derived from the analysis of the clinical trial data. Furthermore, the Independent Ethics Committee of the Hospital Universitario de La Princesa approved the current retrospective observational study (study code: 5709; 2024) and waived the necessity of requesting additional informed consent according to the Spanish Biomedical Law.

### 2.2. Data Collection

Data on the following variables were collected from clinical trial databases: demographic characteristics (e.g., sex, biogeographic origin or ethnicity, age, body surface area [BSA], weight, and height); disease status (tumor type/location [e.g., breast, colon, endometrium, gastric, hepatic, lung, ovary, pancreas, sarcoma, or other] and the Eastern Cooperative Oncology Group Performance Status [ECOG-PS] score); and laboratory measurements (e.g., hematology [hemoglobin blood levels {Hb}, lymphocyte, monocyte, neutrophil, platelet, white blood cell counts, and hematocrit {HCT}]; biochemistry [AAG, albumin, alkaline phosphatase {ALP}, alanine transaminase {ALT}, aspartate transaminase {AST}, direct and total bilirubin, calcium, total cholesterol, creatinine, creatinine kinase {CK}, creatinine clearance, CRP, glucose, lactate dehydrogenase {LDH}, and total protein]; and coagulation [e.g., international normalized ratio {INR}]). The neutrophil-to-lymphocyte (N-L) and the platelet-to-lymphocyte (P-L) ratios were calculated from the collected variables.

Self-reported ethnicity or race was reported as a biogeographic origin, as recommended previously [11], with the intention of reducing the historical–cultural impacts of race/ethnicity variables in favor of a more objective, genetically-based classification (e.g., “White” or “Caucasian” is reported as “European”; “Japanese” as “East Asian”; and “Black” as “Sub-Saharan African”, according to the recruitment site).

### 2.3. Descriptive and Statistical Analyses

For the total study population, the normality of the distributions of the continuous variables was assessed using a Kolmogorov–Smirnov test. For those variables that did not conform to a normal distribution, a logarithmic transformation was applied, and the distribution was reassessed. For univariate analyses, parametric t- or ANOVA tests were conducted, based on the limit central theorem and the large sample size. The mean (standard deviation [SD]) values are provided for the description of the continuous variables. The interdependence among categorical variables was explored through cross-tables (containing count and %) and a Chi-squared or Fisher exact test for statistical inference. No correction for multiple comparisons was conducted based on the descriptive nature of this work. In the training dataset, used for model development, multivariate stepwise regression was conducted for variable selection in each equation. A criterion of biological causality was applied to inform model building. All analyses were run on RStudio 4.3.2 Build 494.

### 2.4. Model Development

Data were split into a training (70%, *n* = 1259) and a validation dataset (30%, *n* = 534) using the *train* function (library: caret) on RStudio. For this step (model development), the training dataset was used; based on the statistical analyses described above, the simulation network was developed. Primary or fixed variables were considered independent, upon which the secondary variables were calculated (i.e., dependent variables). The models were constructed in RStudio with the *lm* function were stepwise, as several primary and secondary variables were interrelated; a hierarchical simulation network was optimized, with the equations becoming increasingly complex at each step. To detect imprecision in these models, different graphs were used to visualize residual errors (i.e., residual vs. residual, residual vs. fitted, normal Q-Q, scale–location, and residuals vs. leverage); in those cases where an assumption of a model was violated (e.g., lack of homoscedasticity or residuals not being homogeneously distributed), logarithmic transformations were implemented. Some of the explored variables were successfully retained in the simulation network, while others were excluded (see the results in Section 3) as no proper fit within the hierarchical simulation network was obtained.

### 2.5. Model Verification

Fixed variables in the training dataset (*n* = 1259) were used to simulate all secondary variables (Table 1) in RStudio using the *predict* function (library: caret). Goodness-of-fit (GOF) plots between the observed and predicted values were created for model verification. Furthermore, the observed and predicted density plots for age, height, body weight, albumin, and AAG serum levels in the training dataset are presented as a visual verification exercise. The data are presented along with data from Cheeti et al.’s work [9] for model comparison; this does not constitute a verification exercise itself but is useful considering the relevance of the latter’s work. Data from Cheeti et al.’s work were extracted using the the WebPlotDigitizer online tool (available online at https://apps.automeris.io/wpd/, accessed on 4 June 2024).

### 2.6. Model Validation

Fixed variables in the validation dataset (*n* = 534) (Table 1) were used to simulate all secondary variables. The GOF plots between the observed and predicted values were created for model validation, in the same fashion as for the model verification.

### 2.7. Model Extrapolation

Several model extrapolation exercises were conducted. Eight cohorts with different percentages of the fixed variables were simulated (Table 1), all of them resembling cohorts of 1200 patients with different characteristics (e.g., only East Asians, only females, and only patients with pancreatic cancer). This exercise was based on the researchers’ predefined criteria, purely exploratory, and designed to demonstrate the ability of the simulation network to create relevant virtual oncology populations. The density plots of the predicted variables are shown in Section 3 (Results).

## 3. Results

### 3.1. Population Characteristics

The baseline and disease characteristics of the study population (training + test datasets, *n* = 1793) are shown in Table 2, stratified by sex because of the clinical relevance of this variable in this context (e.g., only females have ovary cancer). The most prevalent sex was female (63%), with 96% of Europeans, 2% of East Asians, and 2% of Sub-Saharan Africans. Age, height, weight, and BSA were significantly higher in males compared to females (all *p* < 0.0001). The most prevalent cancer type/location was lung cancer (48%), significantly more prevalent in males (83%) compared to females (30%, *p* < 0.0001); males also showed a higher prevalence of pancreatic cancer (7% vs. 4% in females, respectively, *p* < 0.0001).

Supplementary Table S1 shows the mean (SD) values of laboratory variables (i.e., hematology, biochemistry, and coagulation variables) according to sex, biogeographic origin, ECOG-PS score, and tumor type. Summarized as follows are the statistically significant differences in the laboratory variables greater than 20%: AAG according to sex; hematocrit, AAG, CRP, and ASP according to biogeographic origin; lymphocyte, monocyte, neutrophile and white blood cell count, AAG, creatin kinase, LDH and ALK levels, and the N-L and P-L ratios according to ECOG-PS score. For tumor type, several statistically significant differences were observed, notably patients with ovarian cancer had an AAG of around 249.3 mg/dl compared to the mean total value of 153.3 mg/dl (i.e., 63% higher), as well as patients with pancreatic, gastric, or hepatic cancer who had ALK values of 195.2, 169.7, and 164.5 U/L, respectively, compared to the mean total value of 111.3 U/L (i.e., 48 to 75% higher).

### 3.2. Model Development and Verification

For tumor type, sex, race, or the ECOG-PS score (i.e., primary variables), the binomial distribution equation as shown in Equation (1) was used.

*Var_i_* ~ *Binomial*(*pi*,*ni*) (1)

where *Var_i_* is the tumor type, sex, race, or ECOG-PS; *p* is the probability; *n* is the size of the simulation; and *i* refers to each specific *Var_i_*.

Sex (male or female) probability was fixed based on the observed tumor type (breast, endometrial, ovarian, colon, hepatic, gastric, pancreatic, sarcoma, and lung); the probability of the remaining primary variables was randomly chosen.

The predictions for the remaining fixed variables were derived from mathematical modeling. Age was fitted according to differences by sex and tumor type/location using a Weibull distribution, as shown in Equation (2).

*Age* ~ *Weibull*(*α*_*T*,*S*_,*λ*_*T*,*S*_) (2)

where *α* is the shape parameter, *λ* is the scale parameter, *Τ* is the tumor type, and *S* is the sex. Supplementary Table S2 depicts the *α* and *λ* values for four different groups (e.g., males without sarcoma and females with sarcoma tumors showed different parameters).

The remaining variables were modeled based on regression multivariate analyses, as shown in Equation (3).

*Y_i_* = *β*_0_ + *β*_1_*xi*_1_ + *β*_2_*xi*_2_ + *…*
*β*_*n*_*xi*_*n*_ + *ε* (3)

where *Y_i_* is the dependent variable; *β*_0_ is the y-intercept; *β*_1_, *β*_2_, and *β_n_* are the slopes of each explanatory variable; and *ε* is the model’s error term, also known as the residuals, which are shown in the Supplementary Table S3.

**Figure 1:**
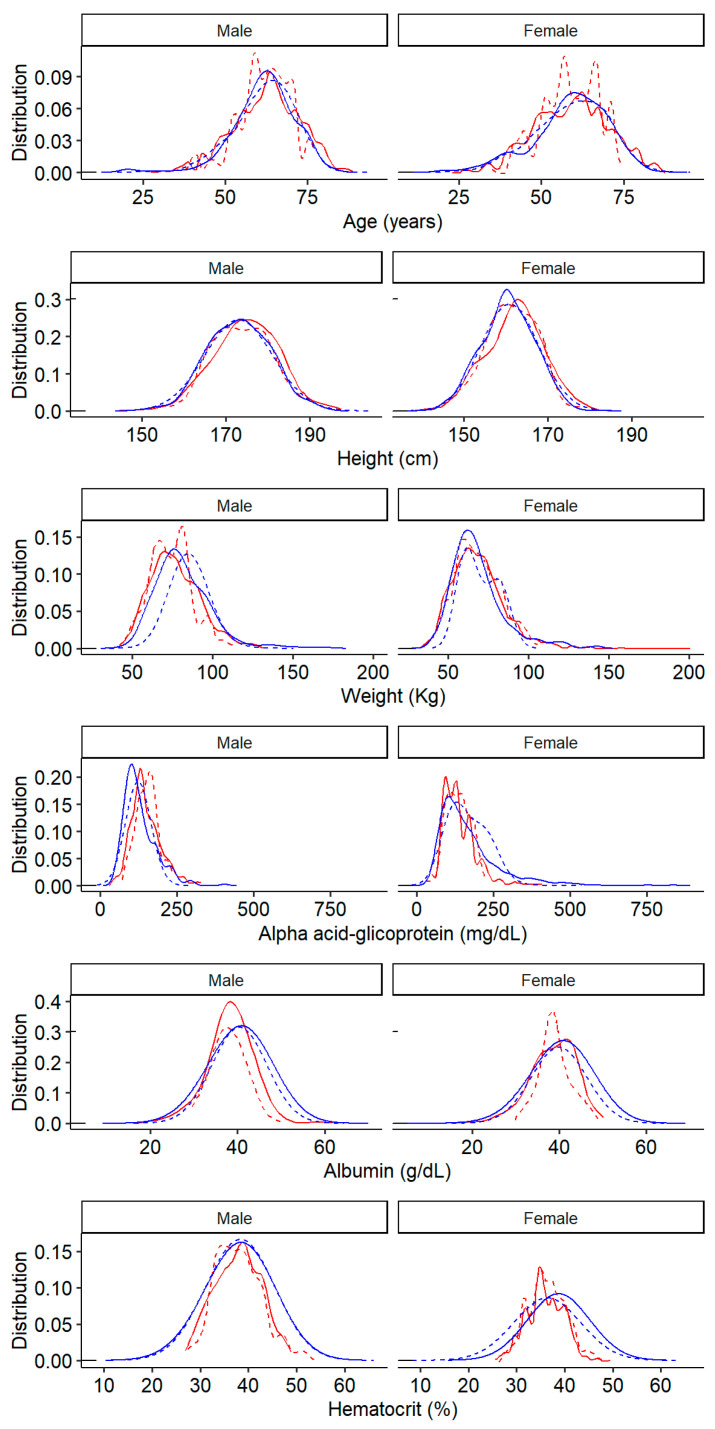
Observed and predicted age, height, body weight, albumin, alpha acid glycoprotein (AAG) serum levels, and hematocrit of the oncology population according to sex. Solid and dashed blue lines represent the observed and predicted data from this study (training dataset), respectively; solid and dashed red lines represent the observed and predicted data from Cheeti et al.’s [9] publication, respectively.

The observed and predicted density curves for age, height, body weight, albumin, AAG serum level, and HTC in the training dataset are shown in Figure 1 and superimposed on those from Cheeti et al.’s publication [9]. The visual comparison of these variables across both study populations revealed no differences in height and body weight. The distribution curves for age (both males and females), AAG, and HTC (only in females) were serrated in Cheeti et al. (i.e., a series of periodically spaced peaks occur), while our data look normally distributed. Finally, the albumin serum levels in males appear slightly lower and less dispersed in Cheeti et al. compared to our study population (Figure 1).

**Figure 2:**
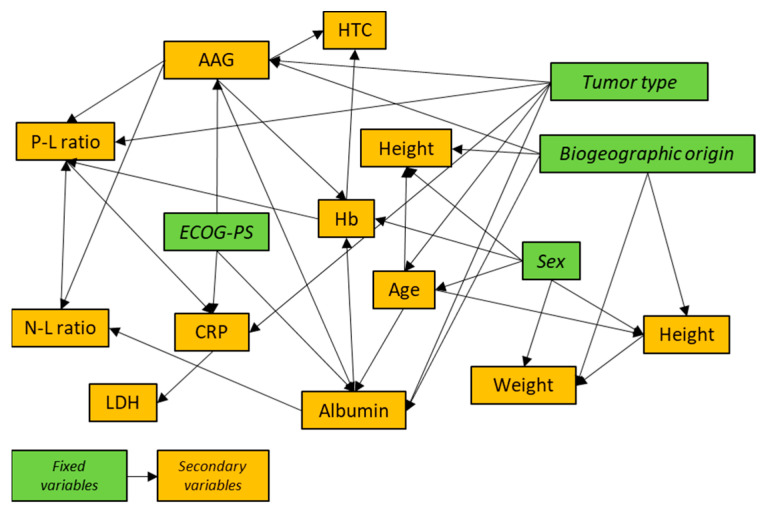
Schematic representation of the simulation network.

Furthermore, Table 3 depicts all of the final modeling equations for the estimation of height, weight, AAG, albumin, Hb, N-L ratio, P-L ratio, CRP, LDH, and HTC, in the same order as developed. The remaining variables could not be satisfactorily modeled. Figure 2 shows a schematic representation of the simulation network. Briefly, height was modeled against age, sex, and biogeographic origin; weight to height, sex, and biogeographic origin; AAG to ECOG-PS, biogeographic origin, and tumor type; albumin to age, AAG, ECOG-PS, race, and tumor type; Hb to albumin, AAG, biogeographic origin, and ECOG-PS; Hb to albumin, AAG, and sex; N-L ratio to AAG and albumin; P-L ratio to Hb, AAG, N-L ratio, and tumor type; CRP to P-L ratio, ECOG-PS, and tumor type; LDH to CRP; and HTC to AAG and Hb.

All equations should account for the model’s error term (*ε*), also known as the residuals, which are provided in Supplementary Table S3. AAG: alpha-1 acid glycoprotein; Hb: hemoglobin; HTC: hematocrit; P-L: platelets/lymphocytes; N-L: neutrophils/lymphocytes; CPR: C-reactive protein; and LDH: lactate dehydrogenase. An interaction between height and female sex was detected in the log weight model, with an estimated coefficient of −0.004; for clarity, this interaction was accounted for in the equation by conditionally including the overall estimate for height–log weight for males (+0.012 * height) and adjusting the overall estimate in females by subtracting the interaction estimate (0.012 − 0.004 = 0.008). Age was fixed following a Weibull distribution.

Supplementary Figure S1 shows GOF plots between the observed and predicted heights, weights, AAG, albumin, Hb, N-L and P-L ratios, CRP, LDH, and HTC (log-transformed when applicable) in the training dataset (model verification). An excellent correlation (R^2^ > 0.9) was observed for HTC (%); a good correlation (R^2^ = 0.4–0.9) was observed for height, P-L ratio, and CRP; a modest correlation (R^2^ = 0.1–0.4) was observed for weight, AAG, albumin, Hb, and LDH; and a poor correlation (R^2^) was observed for the N-L ratio.

An arrow’s direction indicates that a variable was used as an independent variable for the calculation of another dependent variable; green boxes symbolize fixed or primary variables (i.e., those whose value was set and used only as independent variables); and yellow boxes symbolize secondary variables (i.e., those that were calculated based on the equations shown in Table 2). AAG: alpha-1 acid glycoprotein; Hb: hemoglobin; HTC: hematocrit; P-L: platelets/lymphocytes; N-L: neutrophils/lymphocytes; CPR: C-reactive protein; and LDH: lactate dehydrogenase. Age was fixed following a Weibull distribution.

### 3.3. Model Extrapolation

**Figure 3:**
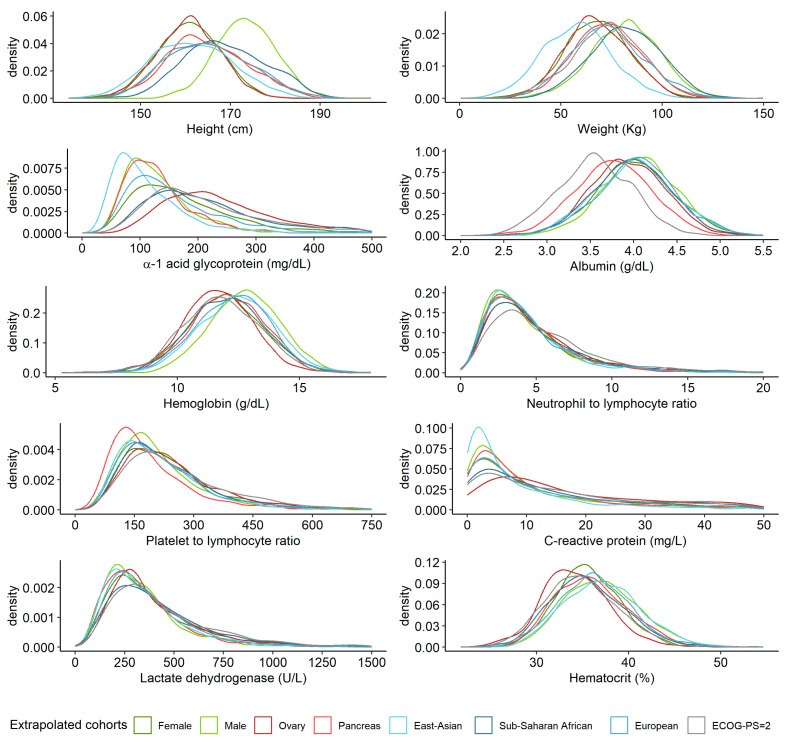
Simulation of height, weight, α-1 acid glycoprotein, albumin, hemoglobin, neutrophil–lymphocyte and platelet–lymphocyte ratios, C-reactive protein, lactate dehydrogenase, and hematocrit in eight different oncology populations of 1200 patients each.

Figure 3 shows the predicted variables of eight simulated cohorts, each of them with 1200 individuals (i.e., East Asians, Sub-Saharan Africans, Europeans, males, females, with an ECOG-PS score of 2, with pancreatic cancer, and with ovarian cancer), where the remaining variables were fixed as in the reference population (i.e., biogeographic origin, sex, ECOG-PS score, and tumor type).

The simulation network predicted a greater height in the cohort of male-only patients compared to the other groups, which included women. A higher weight in the Sub-Saharan African cohort was observed. A higher AAG value was detected in the cohort of females with ovarian cancer, particularly when compared to the East Asians cohort. Patients with ECOG-PS = 2 showed low albumin serum levels, particularly when compared with Sub-Saharan Africans. The Hb level was, along with height, higher in males compared to other cohorts with females. Similar differences were observed for HTC, which was higher in males compared to females with ovarian cancer. CRP was distributed differently across the cohorts, with East Asians having the most patients in the 0–10 mg/L range and few patients above 10 mg/L. In contrast, patients with ovarian cancer had a more flattened distribution, with no peak in the 0–10 mg/L range, and CRP levels spread more evenly from 0 to 40 mg/L. Other smaller differences can be observed in Figure 3.

## 4. Discussion

The demographic and laboratory characteristics of a large, diverse oncology population are extensively described and analyzed in this work. In addition, a simulation network of 4 fixed and 11 secondary variables was optimized, verified, and validated, and the estimation equations are provided.

The study population in this work was somewhat different in age, weight, height, albumin, and AAG compared to that in Cheeti et al.’s work [9]. These differences can be explained, among other causes, by tumor indication, number of lines and type of previous treatments, or geographical location of the clinical trials. These differences encouraged us to include the approach of ethnic diversity in our work. Additionally, were able to incorporate the following variables into the simulation network: Hb, CPR, and LDH serum levels, as well as the N-L and P-L ratios and HTC.

The ability to predict demographic, clinical, or laboratory characteristics/variables of specific patient populations is a valuable tool in clinical research; estimating missing biomarkers prevents excluding patients with useful clinical outcomes; similarly, budget constraints may limit the collection of some variable information, which could be estimated through a simulation network. Furthermore, PBPK models require a population building block, whose demographic, clinical, and laboratory characteristics determine model predictions to a considerable extent. The better this block resembles the population of interest, the greater predictive capacity the model will have. Hence, estimating the clinical and laboratory variables associated with the pharmacokinetics, safety, and efficacy of drugs is crucial to enhance PBPK applications. Below, the relevance of each of the 15 variables in the simulation network is discussed, as well as the observed values in the study population.

### 4.1. Sex

Sex influences cancer incidence, prognosis, and mortality, with males generally having worse outcomes than females due to genetic and environmental factors [12,13,14,15]. Males show higher rates of cancer and related mortality, particularly in Kaposi sarcoma, larynx cancer, mesothelioma, and liver cancers, though they exhibit better survival in Kaposi sarcoma, larynx, and oropharynx cancers [12]. In contrast, females tend to have higher survival rates in cancers like thyroid, salivary gland, breast, gastrointestinal, and skin melanoma [12]. Given its importance and ease of access, sex was set as the primary variable. In this study, males had a significantly higher prevalence of lung and pancreatic cancer, while ovarian, endometrial, and breast cancers were exclusive to females. Serum AAG levels were over 20% higher in males, consistent with previous findings [16].

### 4.2. Performance Status

The ECOG Performance Status (ECOG-PS) scale measures functional impairment in patients with cancer, ranging from 0 (fully active) to 5 (death) [17]. Functional disability is linked to tumor progression and affects survival. While overall survival (OS) is the most reliable clinical endpoint, progression-free survival (PFS) is often used because it requires a smaller sample size and shorter follow-up period, despite its limitations. RECIST defines progressive disease as 20% in the sum of the diameters of up to five target lesions (with a maximum of two lesions per organ), compared to the smallest sum observed during the study [18]. Clinical trials must account for ECOG-PS when designing studies and selecting endpoints, as patients with higher ECOG-PS scores (e.g., 4) have worse survival than those with lower scores (e.g., 0 or 1) [19]. In this study, all patients had ECOG-PS scores equal to or below 2, yet scores between 0 and 2 were associated with over 20% of the variation in variables like AAG and LDH, highlighting the predictive value of ECOG-PS in assessing therapeutic decline.

### 4.3. Tumor Type

In this study, statistically significant differences were found in the ECOG-PS score count according to tumor type; for example, 60% of patients with breast cancer had an ECOG-PS score of 0 and 40% of 1, while these percentages were 40% and 55% in patients with gastric cancer, of which 5% had an ECOG-PS score of 2.

Numerous factors determine the aggressiveness of a malignancy, even within the same tumor type or location. Furthermore, chemotherapy’s effectiveness varies across tumor types. Greater tumor aggressiveness and progression expectedly lead to greater weakening of the performance status, which should be reflected in a higher ECOG-PS score. In this study, we observed great variability in the laboratory variables associated with tumor type and decided to set tumor type as a primary variable. The extent and significance of all of the observed deviations are beyond the scope of this work. However, in view of our results, tumor type and performance status seem clearly interrelated and, therefore, it would be expected to observe pathophysiological variations across tumor types. Considering the capacity of this variable to capture heterogeneity in laboratory measurements, it was designated as a primary or fixed variable. Several differences greater than 20% in the laboratory variables were observed according to tumor type; the finding of elevated AAG serum levels in women with ovarian cancer is noteworthy and consistent with previous reports [20], which suggests that it could be used as a biomarker for early detection of the disease, a hypothesis supported by existing research [21].

### 4.4. Biogeographic Origin

The “biogeographic origin” variable aims to replace the social and cultural aspects of “race” or “ethnicity” with a more objective measure of genetic characteristics, providing better predictive power for differences in drug response [11]. However, some biases remain because the exact biogeographic origin cannot be directly determined and is inferred from lineage and phenotype. When assessing the impact of biogeographic origin on clinical outcomes, height and weight should be considered as covariates due to their variability across populations and their role as independent predictors of drug response.

The following are some examples of different drug responses among populations: (a) azathioprine or 6-mercaptopurine toxicity because of *TPMT* genetic variation (e.g., most Central/South Asians are normal metabolizers [i.e., carry two TPMT*1 alleles], while >7% of Europeans or Latino Americans are intermediate or poor metabolizers due to the high prevalence of the TPMT*3A allele), which is related to dose requirements [22,23]; (b) risk of capecitabine and 5-fluorouracil toxicity because of *DPYD* genetic variation (e.g., DPYD*HapB3 is more prevalent in Central/South Asians and Europeans than in Latino Americans); and (c) risk of irinotecan toxicity because of *UGT1A1* genetic variation (e.g., UGT1A1*28 shows a prevalence of 10–40% across different populations but only of 4% in Oceanians). All of the latter ethnicity-specific differences can be considered pharmacogenetic interactions.

Other genetic differences across populations may condition response to chemotherapy (i.e., cancer genetics). For instance, Shubeck et al. (2023) [24] found significant racial and ethnic differences in pathologic complete response (pCR) rates in patients with stages I–III breast cancer treated with neoadjuvant hemotherapy. These differences were subtype-specific and were found to account for a substantial portion of the identified survival disparity. “Black” patients had lower pCR rates for triple-negative and *HR−/ERBB2+* breast cancer but higher pCR rates for *HR+/ERBB2−* breast cancer, whereas “Asians” and “Pacific Islanders” exhibited higher pCR rates for *HR−/ERBB2+* malignancies. Furthermore, the *ERBB2* copy number could account for some of these within-subtype disparities.

### 4.5. Age

Age serves as a proxy for variations in numerous physiological and pathophysiological processes. Differences between newborns and adults due to incomplete neonatal development and the effects of aging significantly influence drug pharmacokinetics and pharmacodynamics. Aging typically results in reduced hepatic and renal clearances, increased volume of distribution, and prolonged half-lives of lipophilic drugs. Additionally, sensitivity to drugs, such as anticoagulants, psychotropics, and cardiovascular medications, often increases with age [25]. Age also impacts laboratory variables, which can affect drug pharmacokinetics. Furthermore, cancer prevalence is age-dependent; for instance, in the United States, in 2009, the incidence of invasive cancer was highest in the 65–69 age group, with a distribution skewed toward older patients [26].

### 4.6. Weight and Height

Weight is a significant predictor of drug pharmacokinetics, reflecting not only an individual’s size and plasma volume but also their body fat percentage, which influences drug bioavailability and distribution. Height, on the other hand, predicts body size rather than body fat percentage. Combining weight and height provides metrics like body mass index (BMI = weight/height^2^) and body surface area (BSA = √ (height × weight/3600)). These metrics help categorize patients as underweight, overweight, or obese (or with low, normal, or high BSA), which can significantly impact pharmacokinetics and dosing requirement [27]. Notably, weight loss is a common side effect of cancer treatments [28], making its modeling crucial in this population.

### 4.7. Plasma Proteins

Plasma is constituted by >90% water and <10% proteins, with a concentration of around 60–80 mg/mL (6–8 g/dL). Of the latter, >50% are albumins and 40% are globulins such as IgG [27]. AAG, also known as orosomucoid, accounts for around 3% of plasma proteins. Drug binding to albumin and AAG affects drug distribution, drug unbound fraction, accumulation at the site of action, and elimination/drug extraction [29]. In the early stage of cancer, albumin levels are normal or slightly low, and they significantly drop when the disease progresses [30]. In this study, no differences in albumin levels were observed based on ECOG-PS scores, likely because only patients with scores below 2 were included; additionally, extremely low albumin levels were excluded based on the clinical trials’ inclusion and exclusion criteria. Therefore, additional research is needed to investigate and model albumin levels in patients with ECOG-PS scores above 2. Conversely, AAG levels are reported to increase with cancer progression [31], which aligns with the observed values of 226 mg/dL in patients with an ECOG-PS = 2 compared to 156 mg/dL and 144 mg/dL in patients with an ECOG-PS = 1 and 0, respectively, and an expected range of 50–130 mg/dL in healthy adults [32].

### 4.8. Hematocrit and Hemoglobin

The blood-to-plasma ratio (R_b_) is calculated by dividing a drug’s blood concentration, C_b_, by its concentration in plasma, C_p_ (R_b_ = C_b_/C_p_). High R_b_ values (e.g., 2 for butorphanol) imply the accumulation of the drug in erythrocytes, while low R_b_ ratios (e.g., 0.5 for doripenem) imply that the drug barely enters the erythrocytes [33]. Hematocrit (HTC) is the volume percentage of erythrocytes in blood. A higher HTC is related to a higher R_b_. Although C_b_ may remain unchanged with a higher HTC or R_b_, the C_p_ is reduced, decreasing the drug’s availability in the target tissues; conversely, a lower HTC or R_b_ can lead to a higher risk of adverse drug reactions [34] because of the higher C_p_.

The direct effects of Hb levels on drug pharmacokinetics are less clear. One study recommended vancomycin dosing based on Hb levels in patients with sepsis [35], although these claims have been contested [36]. Nonetheless, it is expected that Hb levels have the same effect as HTC, since both variables are correlated [37] and proxy variables of R_b_.

### 4.9. Lactate Dehydrogenase

Elevated serum lactate dehydrogenase (LDH) levels are associated with adverse prognoses and poor treatment outcomes in various cancers. However, interpreting LDH levels can be complex, as they are linked to the activation of oncogenic signaling pathways, metabolic activity, invasiveness, and immunogenicity of tumors [38]. In this study, similar to AAG levels, LDH levels were significantly higher in patients with greater ECOG-PS scores—613 U/L for ECOG-PS = 2; 409 U/L for ECOG-PS = 1; and 343 U/L for ECOG-PS = 0—compared to the reference range of 140–280 U/L in healthy adults [39]. The LDH modeling may have limited value for pharmacokinetic predictions; however, it could serve as a marker for oncologic disease progression or as a surrogate marker for the pharmacodynamics (effectiveness) of chemotherapeutic drugs.

### 4.10. Inflammation Biomarkers

CRP is a mediator and indicator of inflammatory processes, including those in patients with cancer. However, its significance for diagnosing disease progression or remission has been challenged. [40]. In this study, a trend toward higher CRP levels was observed with increasing ECOG-PS scores—30 mg/L for ECOG-PS = 2; 26 mg/L for ECOG-PS = 1; and 23 mg/L for ECOG-PS = 0—compared to the expected range of 0.02–13.5 mg/L in healthy adults [41]. Inflammation can modify the activity and/or expression of drug-metabolizing enzymes and transporters, including CYP1A, CYP3A, CYP2B, CYP2C, and UGT1A1 enzymes and SLC22A4, SLCO4A1, SLCO2B1, ABCB1, and ABCG2 transporters [42]. Similarly, the P-L ratio is a biomarker for inflammatory processes [43]; here, the P-L ratio was higher in women compared to men and related to the ECOG-PS score in the same way as for CRP. In contrast, baseline inflammation is reportedly higher in men than in women [44]; considering that other inflammatory biomarkers such as CRP did not differ between men and women, and in light of the borderline observed p-value (*p* = 0.030), this association may be spurious. The N-L ratio is also considered an inflammatory biomarker and prognostic biomarker of various diseases [45]; here, an association between the N-L ratio and the ECOG-PS score was also observed.

### 4.11. Model Extrapolation

Eight oncology populations with diverse demographic and clinical characteristics were effectively simulated. Variations in the clinical and laboratory variables among the simulated groups demonstrated the predictive capability of the simulation network based on primary variables, with the distribution of variables aligning with the expected outcomes. The successful model extrapolation confirms that these simulations are now ready for further application in clinical and research settings.

Incorporating these populations into freely accessible PBPK models will be crucial in the coming years for ensuring equity and equality in drug development. This approach aligns with FDA initiatives, such as the “Enhancing the Diversity of Clinical Trial Populations” guidance [46] and the “CDER Patient-Focused Drug Development” approach [47], which emphasize the need for inclusive clinical research to reflect diverse patient populations, incorporating the variability across different demographic groups into drug development. It also endorses FDA’s “Project Optimus”, which aims to reform the dose optimization paradigm in oncology [48]. By predicting pharmacokinetics, safety, and efficacy in underrepresented populations, this strategy enhances accessibility and helps prevent discrimination. Optimizing drug development in this manner ensures that treatments are safe and effective for patients across all regions and irrespective of biogeographic origins.

In summary, this network constitutes a valuable tool to simulate patient’s relevant characteristics/variables, which may be useful for estimating pharmacokinetics in virtual oncology populations and for model-based optimization of oncology treatments.

### 4.12. Limitations

The oncology population in this study was derived from clinical trials involving specific oncology groups. Consequently, this simulation network is designed to replicate the demographic and laboratory parameters of patients within the variability ranges of our population. Simulations that significantly deviate from these validation ranges may lack accuracy, for instance, simulations of a pediatric population or patients with severe anorexia, since age and weight would be fixed outside the acceptable ranges. This limitation also applies to the other fixed parameters (e.g., tumor type, biogeographic origin, and ECOG-PS score). Nevertheless, the sample size was sufficiently large, and the patient characteristics were varied enough to allow the network to effectively simulate most adult oncology patients. Similar studies are needed to optimize simulation networks capable of predicting special oncology populations and oncology patients with other biogeographic origins, providing a more comprehensive representation of the diverse patient populations encountered in oncology.

## 5. Conclusions

The demographic and laboratory characteristics of a large, diverse oncology population were extensively described and analyzed. A simulation network of 4 fixed and 11 secondary variables was optimized, verified, and validated, and the estimation equations provided to ensure reproducibility in future studies. A simulation exercise of eight oncology populations was carried out to demonstrate an example of the usefulness of this network. Open access to the equations is provided.

## Figures and Tables

**Table 1:** Fixed primary variables in the reference training dataset and in the extrapolated cohorts.

Cohort	Biogeographic Origin			Sex		ECOG-PS			Tumor Type								
	E	SSA	EA	M	F	0	1	2	Br	Co	End	Gas	Hep	Lu	Ov	Pa	Sar
Reference Population	96%	2%	2%	37%	63%	35%	55%	5%	7%	1%	7%	1%	1%	48%	28%	4%	3%
1. Only East Asians	0%	0%	100%														
2. Only Sub-Saharan Africans	0%	100%	0%														
3. Only Europeans	100%	0%	0%														
4. Only males				100%	0%				0%	2%	0%	2%	3%	76%	0%	11%	6%
5. Only females				0%	100%				10%	2%	10%	1%	1%	30%	40%	3%	3%
6. Only ECOG-PS = 2						0%	0%	100%									
7. Only pancreatic cancer									0%	0%	0%	0%	0%	0%	0%	100%	0%
8. Only ovarian cancer				0%	100%				0%	0%	0%	0%	0%	0%	100%	0%	0%

Shaded wells indicate the same percentage as in the “reference population”, which corresponds to the training dataset (*n* = 1259). Unshaded wells indicate that a fixed variable changed compared to the “reference population”. Cohorts 1–8 (all *n* = 1200) were extrapolated using the simulation network and the % of fixed variables as shown in the table. E: Europeans; SSA: Sub-Saharan African; EA: East Asian; M: male; F: female; Br: breast; Co: colon; End: endometrium; Gas: gastric; Hep: hepatic; Lu: lung; Ov: ovary; Pa: pancreas; Sar: sarcoma.

**Table 2:** Baseline and disease characteristics of the study population.

Variable		Male (*n* = 615)	Female (*n* = 1178)	Total (*n* = 1793)
Demographic characteristics	Age (years [mean and SD])	62.3 (9.3) *	59.1 (11.2)	60.2 (10.7)
	Height (cm [mean and SD])	173.0 (7.5) *	160.7 (6.4)	165.0 (9.0)
	Weight (kg [mean and SD])	81.0 (17.1) *	68.2 (16.2)	72.6 (17.6)
	BSA (kg/cm [mean and SD])	1.9 (0.2) *	1.7 (0.2)	1.8 (0.2)
Tumor type/location (count and %)	Breast (%)	0 (0%)	128 (10.9)	128 (7.1)
	Colon (%)	12 (2.0)	10 (0.8)	22 (1.2)
	Endometrial (%)	NA	120 (10.2)	120 (6.7)
	Gastric (%)	5 (0.8)	2 (0.2)	7 (0.4)
	Hepatic (%)	9 (1.5)	7 (0.6)	16 (0.9)
	Lung (%)	513 (83.4) *	352 (29.9)	865 (48.2)
	Ovarian (%)	NA	499 (42.4)	499 (27.8)
	Pancreatic (%)	43 (7.0) *	23 (2.0)	66 (3.7)
	Sarcoma (%)	33 (5.4)	37 (3.1)	70 (3.9)
ECOG-PS (score)	0	217 (35.3)	529 (44.9)	746 (41.6)
	1	382 (62.1)	620 (52.6)	1002 (55.9)
	2	16 (2.6)	29 (2.5)	45 (2.5)
Biogeographical origin (count and %)	Japanese (East Asian)	4 (1%)	23 (2%)	27 (1%)
	Black (Sub-Saharan African)	6 (1%)	22 (2%)	28 (2%)
	White (European)	605 (98%)	1133 (96%)	1738 (97%)

NA: not applicable. The percentage in the “male” and “female” columns represents the count in each sex according to the total of each tumor type, ECOG-PS score, or biogeographic origin. The percentage in the “total” column represents the total count according to the total number of patients. * *p* < 0.05 after *t*-test or Chi-squared test. No statistical test was performed for cells with an NA (not applicable) value.

**Table 3:** Linear regression equations and order of the estimations of the calculated variables.

Order	Equation
**1**	**Height (cm)** = 179.9 − 0.16 * Age (years) − 12.81 if Female + 0.00 if Male + 6.42 if Sub-Saharan African + 3.83 if European + 0.00 if East Asian
**2**	**Log weight (log Kg)** = 2.10 + 0.012 * Height (cm) if Male + 0.008 * Height (cm) if Female + 0.562 if Female + 0.00 if Male + 0.262 if Sub Saharan African + 0.144 if European + 0.00 if East Asian
**3**	**Log AAG (log mg/dL)** = 4.32 + 0.161 if ECOG-PS = 1 + 0.449 if ECOG-PS = 2 + 0.000 if ECOG-PS = 0 + 0.634 if Sub Saharan African + 0.370 if European + 0.00 if East Asian + 0.622 if Ovarian Tumor + 0.000 if Another Tumor
**4**	**Albumin (g/dL)** = 5.546 − 0.004 * Age (years) − 0.292 * Log AAG (log mg/dL) − 0.128 if ECOG-PS = 1 − 0.429 if ECOG-PS = 2 + 0.000 if ECOG-PS = 0 + 0.276 if Sub Saharan African + 0.248 if European + 0.00 if East Asian − 0.339 if Pancreatic, Hepatic, or Gastric Tumor + 0.000 if Another Tumor
**5**	**Hb (g/dL)** = 12.35 + 0.575 * Albumin (g/dL) − 0.793 * Log AAG (log mg/dL) − 0.680 if Female + 0.000 if Male
**6**	**Log N-L ratio** = 1.47 − 0.0073 * Log AAG (log mg/dL) − 0.16 * Albumin (g/dL)
**7**	**Log P-L ratio** = 5.52 − 0.057 * Hb (g/dL) + 0.0016 * AAG (mg/dL) + 0.0622 * N-L Ratio − 0.204 if Pancreatic, Hepatic, or Gastric Cancer + 0.000 if Another Tumor
**8**	**Log CRP (log mg/L)** = 1.394 + 0.002 * P-L Ratio + 0.368 if ECOG-PS = 1 + 0.598 if ECOG-PS = 2 + 0.000 if ECOG-PS = 0 − 2.22 if Breast Cancer + 0.000 if Another Tumor
**9**	**Log LDH (log U/L)** = 5.709 + 0.001 * CRP (mg/L)
**10**	**Log HTC (%)** = 5.93 + 0.04 * Log AAG (log mg/dL) + 2.515 * Hb (g/dL)

## Data Availability

Data used for this work are confidential and may be available upon reasonable request from the sponsor through the corresponding author.

## Supplementary Materials

The following supporting information can be downloaded at: https://www.mdpi.com/article/10.3390/pharmaceutics16121548/s1, Table S1: Laboratory parameters according to (a) sex; (b) biogeographic origin; (c) ECOG-PS score; (d) tumor type; Table S2: Summary of the Weibull parameters for age distributions in four groups; Table S3: Summary of model residuals; Figure S1: Model verification goodness-of-fit plots; Figure S2: Model validation goodness-of-fit plots.

## References

[1] Garattini S. (2007). Pharmacokinetics in cancer chemotherapy. Eur. J. Cancer.

[2] Lankelma J. (2002). Tissue transport of anti-cancer drugs. Curr. Pharm. Des..

[3] Woo J., Chan H.S., Or K.H., Arumanayagam M. (1994). Effect of age and disease on two drug binding proteins: Albumin and alpha-1- acid glycoprotein. Clin. Biochem..

[4] Kremer M., Wilting J., Janssen L.H. (1988). Drug binding to human alpha-1-acid glycoprotein in health and disease. Pharmacol. Rev..

[5] Piafsky K.M. (1980). Disease-induced changes in the plasma binding of basic drugs. Clin. Pharmacokinet..

[6] Israili Z.H., Dayton P.G. (2001). Human alpha-1-glycoprotein and its interactions with drugs. Drug Metab. Rev..

[7] Teutonico D., Musuamba F., Maas H.J., Facius A., Yang S., Danhof M., Della Pasqua O. (2015). Generating Virtual Patients by Multivariate and Discrete Re-Sampling Techniques. Pharm. Res..

[8] Gerhart J.G., Carreno F.O., Edginton A.N., Sinha J., Perrin E.M., Kumar K.R., Rikhi A., Hornik C.P., Harris V., Ganguly S. (2022). Development and Evaluation of a Virtual Population of Children with Obesity for Physiologically Based Pharmacokinetic Modeling. Clin. Pharmacokinet..

[9] Cheeti S., Budha N.R., Rajan S., Dresser M.J., Jin J.Y. (2013). A physiologically based pharmacokinetic (PBPK) approach to evaluate pharmacokinetics in patients with cancer. Biopharm. Drug Dispos..

[10] Yasuda S.U., Zhang L., Huang S.M. (2008). The role of ethnicity in variability in response to drugs: Focus on clinical pharmacology studies. Clin. Pharmacol. Ther..

[11] Huddart R., Fohner A.E., Whirl-Carrillo M., Wojcik G.L., Gignoux C.R., Popejoy A.B., Bustamante C.D., Altman R.B., Klein T.E. (2019). Standardized Biogeographic Grouping System for Annotating Populations in Pharmacogenetic Research. Clin. Pharmacol. Ther..

[12] Dong M., Cioffi G., Wang J., Waite K.A., Ostrom Q.T., Kruchko C., Lathia J.D., Rubin J.B., Berens M.E., Connor J. (2020). Sex Differences in Cancer Incidence and Survival: A Pan-Cancer Analysis. Cancer Epidemiol. Biomarkers Prev..

[13] Siegel R.L., Miller K.D., Jemal A. (2019). Cancer statistics, 2019. CA Cancer J. Clin..

[14] Goldman N., Glei D.A., Weinstein M. (2016). What Matters Most for Predicting Survival? A Multinational Population-Based Cohort Study. PLoS ONE.

[15] Yang W., Warrington N.M., Taylor S.J., Whitmire P., Carrasco E., Singleton K.W., Wu N., Lathia J.D., Berens M.E., Kim A.H. (2019). Sex differences in GBM revealed by analysis of patient imaging, transcriptome, and survival data. Sci. Transl. Med..

[16] Kishino S., Nomura A., Itoh S., Nakagawa T., Takekuma Y., Sugawara M., Furukawa H., Todo S., Miyazaki K. (2002). Age- and gender-related differences in carbohydrate concentrations of alpha1-acid glycoprotein variants and the effects of glycoforms on their drug-binding capacities. Eur. J. Clin. Pharmacol..

[17] Azam F., Latif M.F., Farooq A., Tirmazy S.H., AlShahrani S., Bashir S., Bukhari N. (2019). Performance Status Assessment by Using ECOG (Eastern Cooperative Oncology Group) Score for Cancer Patients by Oncology Healthcare Professionals. Case Rep. Oncol..

[18] Villaruz L.C., Socinski M.A. (2013). The clinical viewpoint: Definitions, limitations of RECIST, practical considerations of measurement. Clin. Cancer Res..

[19] Assayag J., Kim C., Chu H., Webster J. (2023). The prognostic value of Eastern Cooperative Oncology Group performance status on overall survival among patients with metastatic prostate cancer: A systematic review and meta-analysis. Front. Oncol..

[20] Choi J.W., Jeong K.H., You J.W., Lee J.W., Moon B.I., Kim H.J., Kim H.J. (2020). Serum Levels and Glycosylation Changes of Alpha-1-Acid Glycoprotein According to Severity of Breast Cancer in Korean Women. J. Microbiol. Biotechnol..

[21] Qiong L., Yin J. (2022). Characterization of alpha-1-acid glycoprotein as a potential biomarker for breast cancer. Bioengineered.

[22] Relling M.V., Schwab M., Whirl-Carrillo M., Suarez-Kurtz G., Pui C.H., Stein C.M., Moyer A.M., Evans W.E., Klein T.E., Antillon-Klussmann F.G. (2019). Clinical Pharmacogenetics Implementation Consortium Guideline for Thiopurine Dosing Based on TPMT and NUDT15 Genotypes: 2018 Update. Clin. Pharmacol. Ther..

[23] Casajús A., Zubiaur P., Méndez M., Campodónico D., Gómez A., Navares-Gómez M., Villapalos-García G., Soria-Chacartegui P., Novalbos J., Román M. (2022). Genotype-Guided Prescription of Azathioprine Reduces the Incidence of Adverse Drug Reactions in TPMT Intermediate Metabolizers to a Similar Incidence as Normal Metabolizers. Adv. Ther..

[24] Shubeck S., Zhao F., Howard F.M., Olopade O.I., Huo D. (2023). Response to Treatment, Racial and Ethnic Disparity, and Survival in Patients With Breast Cancer Undergoing Neoadjuvant Chemotherapy in the US. JAMA Netw. Open.

[25] Mangoni A.A., Jackson S.H. (2004). Age-related changes in pharmacokinetics and pharmacodynamics: Basic principles and practical applications. Br. J. Clin. Pharmacol..

[26] White M.C., Holman D.M., Boehm J.E., Peipins L.A., Grossman M., Henley S.J. (2014). Age and cancer risk: A potentially modifiable relationship. Am. J. Prev. Med..

[27] Green B., Duffull S.B. (2004). What is the best size descriptor to use for pharmacokinetic studies in the obese?. Br. J. Clin. Pharmacol..

[28] Hager K.K. (2016). Management of Weight Loss in People With Cancer. J. Adv. Pract. Oncol..

[29] Larsen M.T., Kuhlmann M., Hvam M.L., Howard K.A. (2016). Albumin-based drug delivery: Harnessing nature to cure disease. Mol. Cell Ther..

[30] Gupta D., Lis C.G. (2010). Pretreatment serum albumin as a predictor of cancer survival: A systematic review of the epidemiological literature. Nutr. J..

[31] Jackson P.R., Tucker G.T., Woods H.F. (1982). Altered plasma drug binding in cancer: Role of alpha 1-acid glycoprotein and albumin. Clin. Pharmacol. Ther..

[32] Trainor G.L. (2007). The importance of plasma protein binding in drug discovery. Expert. Opin. Drug Discov..

[33] Mamada H., Iwamoto K., Nomura Y., Uesawa Y. (2021). Predicting blood-to-plasma concentration ratios of drugs from chemical structures and volumes of distribution in humans. Mol. Divers..

[34] Piletta-Zanin A., De Mul A., Rock N., Lescuyer P., Samer C.F., Rodieux F. (2021). Case Report: Low Hematocrit Leading to Tacrolimus Toxicity. Front. Pharmacol..

[35] Chuma M., Makishima M., Imai T., Tochikura N., Suzuki S., Kuwana T., Sawada N., Iwabuchi S., Sekimoto M., Nakayama T. (2019). Relationship between hemoglobin levels and vancomycin clearance in patients with sepsis. Eur. J. Clin. Pharmacol..

[36] Deenen M.J., Ter Heine R. (2019). Is vancomycin clearance really correlated with hemoglobin? Arguments that it’s not. Eur. J. Clin. Pharmacol..

[37] Flor C.R., Baldoni A.O., Garcia Mateos S.O., Sabino E.C., Oliveira C.D.L. (2023). Comparison of Two Methods of Capillary Sampling in Blood Pre-Donation Anemia Screening in Brazil. Hematol. Rep..

[38] Claps G., Faouzi S., Quidville V., Chehade F., Shen S., Vagner S., Robert C. (2022). The multiple roles of LDH in cancer. Nat. Rev. Clin. Oncol..

[39] Farhana A., Lappin S.L. (2024). Biochemistry, Lactate Dehydrogenase.

[40] Hart P.C., Rajab I.M., Alebraheem M., Potempa L.A. (2020). C-Reactive Protein and Cancer-Diagnostic and Therapeutic Insights. Front. Immunol..

[41] Kindmark C.O. (1972). The concentration of C-reactive protein in sera from healthy individuals. Scand. J. Clin. Lab. Invest..

[42] Stanke-Labesque F., Gautier-Veyret E., Chhun S., Guilhaumou R. (2020). French Society of Pharmacology and Therapeutics. Inflammation is a major regulator of drug metabolizing enzymes and transporters: Consequences for the personalization of drug treatment. Pharmacol. Ther..

[43] Gasparyan A.Y., Ayvazyan L., Mukanova U., Yessirkepov M., Kitas G.D. (2019). The Platelet-to-Lymphocyte Ratio as an Inflammatory Marker in Rheumatic Diseases. Ann. Lab. Med..

[44] Martinez de Toda I., Gonzalez-Sanchez M., Diaz-Del Cerro E., Valera G., Carracedo J., Guerra-Perez N. (2023). Sex differences in markers of oxidation and inflammation. Implications for ageing. Mech. Ageing Dev..

[45] Durmus E., Kivrak T., Gerin F., Sunbul M., Sari I., Erdogan O. (2015). Neutrophil-to-Lymphocyte Ratio and Platelet-to-Lymphocyte Ratio are Predictors of Heart Failure. Arq. Bras. Cardiol..

[46] Food and Drug Administration, U.S. (2020). Enhancing the Diversity of Clinical Trial Populations—Eligibility Criteria, Enrollment Practices, and Trial Designs Guidance for Industry.

[47] Food and Drug Administration, U.S. (2024). CDER Patient-Focused Drug Development.

[48] Food and Drug Administration, U.S. (2024). Project Optimus.

