# Supplementary material for: Description and Modeling of Relevant Demographic and Laboratory Variables in a Large Oncology Cohort to Generate Virtual Populations
Source: Pharmaceutics. 2024 Dec 3;16(12):1548. doi: 10.3390/pharmaceutics16121548 (PMC11728769; doi:10.3390/pharmaceutics16121548)
Supplement: Supplementary file 1 [file pharmaceutics-16-01548-s001.zip › pharmaceutics-3265576-supplementary.pdf]

Supplement to: Description and modeling of relevant demographic and laboratory parameters in a large oncology cohort to generate virtual populations

Supplementary Tables

Supplementary Table S1. Laboratory parameters according to a) sex, b) biogeographic origin. c) ECOG-PS score and d) tumor type. 2
Supplementary Table S2. Summary of the Weibull parameters for age distributions in four groups. 9
Supplementary Table S3. Summary of model residuals. 10

Supplementary Figures

Supplementary Figure 1. Model verification goodness of fit plots. 11
Supplementary Figure 2. Model validation goodness of fit plots. 12

Supplementary Table S1. Laboratory variables according to a) sex, b) biogeographic origin. c) ECOG-PS score and d) tumor type.

a)

Parameter	Male (n=615)	Female (n=1178)	Total (n=1793)	p
Hematocrit (%)	35.2 (10.3)	37.5 (3.2)	36.2 (8.2)	<0.0001
N	30	37	67	
Hemoglobin (g/dL) (SD)	12.8 (1.6)	12.0 (1.4)	12.3 (1.5)	<0.0001
N	578	1152	1730	
Lymphocytes (count*10^9) (SD)	1.4 (0.9)	1.4 (0.8)	1.4 (0.8)	0.0667
N	576	1151	1727	
Monocytes (count*10^9) (SD)	0.7 (0.3)	0.5 (0.3)	0.6 (0.3)	<0.0001
N	155	738	893	
Neutrophils (count*10^9) (SD)	5.4 (3.6)	4.7 (2.4)	4.9 (2.9)	<0.0001
N	578	1151	1729	
Platelets (count*10^9) (SD)	245.6 (94.4)	270.3 (102.0)	262.0 (100.2)	<0.0001
N	578	1152	1730	
White blood cells (count*10^9) (SD)	7.5 (2.8)	6.8 (2.7)	7.0 (2.7)	<0.0001
N	578	1152	1730	
Alpha-1 acid glycoprotein (mg/dL) (SD)	128.4 (51.4)	171.2 (103.9)	156.4 (91.5)	<0.0001
N	390	734	1124	
Albumin (g/dL) (SD)	4.1 (0.4)	4.0 (0.4)	4.0 (0.4)	0.3348
N	578	1152	1730	
Alkaline phosphatase (U/L) (SD)	104.7 (79.5)	108.6 (70.8)	107.3 (73.8)	0.3063
N	573	1149	1722	
Alkaline transaminase (U/L) (SD)	26.0 (19.3)	22.7 (15.0)	23.8 (16.6)	0.0001
N	578	1152	1730	
Aspartate transaminase (U/L) (SD)	26.0 (16.1)	25.7 (16.7)	25.8 (16.5)	0.7669
N	575	1150	1725	
Direct bilirrubin (mg/dL) (SD)	0.2 (0.6)	0.2 (0.1)	0.2 (0.4)	0.0819
N	257	452	709	
Total bilirubin (mg/dL) (SD)	0.5 (0.2)	0.4 (0.2)	0.5 (0.2)	<0.0001
N	578	1152	1730	
Calcium (mmol/L) (SD)	2.4 (0.5)	2.4 (0.1)	2.4 (0.3)	0.0851
N	571	742	1313	
Total cholesterol (mg/dL) (SD)	179.6 (44.0)	203.0 (43.3)	195.0 (44.9)	<0.0001
N	417	798	1215	
Creatinine kinase (U/L) (SD)	74.9 (53.1)	69.4 (43.7)	71.2 (47.1)	<0.0001
N	565	1128	1693	
Creatinine (mg/dL) (SD)	0.9 (0.2)	0.8 (0.2)	0.8 (0.2)	<0.0001
N	578	1152	1730	
Creatinine clearance (mL/min) (SD)	98.6 (37.5)	87.3 (32.9)	91.3 (35.0)	<0.0001

N	438	809	1247	
C-reactive protein (mg/L) (SD)	25.3 (47.1)	27.4 (104.5)	26.4 (83.5)	0.6822
N	486	587	1073	
Glucose (mmol/L)	6.5 (2.7)	5.9 (1.9)	6.1 (2.2)	<0.0001
N	575	1147	1722	
Lactate dehydrogenase (U/L)	357.6 (296.7)	389.9 (321.6)	375.7 (311.2)	0.0635
N	570	726	1296	
Total protein (g/dL)	7.1 (0.6)	7.1 (0.6)	7.1 (0.6)	0.6291
N	285	924	1209	
INR (SD)	1.1 (0.2)	1.0 (0.2)	1.1 (0.2)	0.0771
N	284	517	801	
N-L ratio	4.9 (3.9)	4.3 (4.1)	4.5 (4.1)	0.0055
N	576	1151	1727	
P-L ratio	225.8 (163.5)	239.7 (173.6)	235.1 (170.4)	0.1090
N	576	1151	1727	

b)

Parameter	East-Asian (n=27)	Sub-Saharan African (n=28)	European (n=1738)	p
Hematocrit (%)	37.7 (4.4)	45.9 (2.1)	35.0 (9.9)	<0.0001
N	10	3	54	
Hemoglobin (g/dL) (SD)	11.6 (1.6)	11.4 (1.4)	12.3 (1.5)	0.0005
N	27	27	1676	
Lymphocytes (count*10^9) (SD)	1.3 (0.5)	1.3 (0.5)	1.4 (0.8)	0.7216
N	27	27	1673	
Monocytes (count*10^9) (SD)	0.5 (0.2)	0.6 (0.3)	0.6 (0.3)	0.7507
N	24	21	848	
Neutrophils (count*10^9) (SD)	4.2 (1.6)	4.8 (2.6)	5.0 (2.9)	0.4025
N	27	27	1675	
Platelets (count*10^9) (SD)	259.0 (112.5)	289.2 (106.2)	261.6 (99.9)	0.3606
N	27	27	1676	
White blood cells (count*10^9) (SD)	6.3 (2.0)	6.9 (2.7)	7.0 (2.8)	0.3452
N	27	27	1676	
Alpha-1 acid glycoprotein (mg/dL) (SD)	100.8 (65.1)	222.0 (174.7)	156.3 (89.1)	0.0001
N	21	20	1083	
Albumin (g/dL) (SD)	3.9 (0.6)	3.9 (0.5)	4.0 (0.4)	0.0264
N	27	27	1676	
Alkaline phosphatase (U/L) (SD)	191.0 (148.2)	110.0 (47.3)	105.9 (71.6)	<0.0001
N	27	27	1668	
Alkaline transaminase (U/L) (SD)	25.1 (17.2)	24.6 (20.8)	23.8 (16.6)	0.8908
N	27	27	1676	

Aspartate transaminase (U/L) (SD)	28.3 (14.2)	39.6 (54.5)	25.5 (15.1)	<0.0001
N	27	27	1671	
Direct bilirrubin (mg/dL) (SD)	0.1 (0.1)	0.2 (0.1)	0.2 (0.4)	0.8349
N	15	9	685	
Total bilirubin (mg/dL) (SD)	0.4 (0.2)	0.4 (0.2)	0.5 (0.2)	0.3953
N	27	27	1676	
Calcium (mmol/L) (SD)	2.3 (0.1)	2.4 (0.1)	2.4 (0.4)	0.6930
N	22	19	1272	
Total cholesterol (mg/dL) (SD)	168.2 (20.8)	156.0 (29.8)	195.7 (44.9)	0.0220
N	4	8	566	
Creatinine kinase (U/L) (SD)	74.0 (41.8)	96.5 (69.6)	70.8 (46.7)	0.0209
N	27	26	1640	
Creatinine (mg/dL) (SD)	0.7 (0.2)	1.0 (0.3)	0.8 (0.2)	0.0003
N	27	27	1676	
Creatinine clearance (mL/min) (SD)	97.5 (38.5)	90.3 (44.1)	91.2 (34.8)	0.7100
N	21	19	1207	
C-reactive protein (mg/L) (SD)	12.4 (21.1)	30.1 (23.9)	26.6 (84.5)	0.7769
N	17	10	1046	
Glucose (mmol/L)	6.8 (2.7)	6.8 (2.5)	6.1 (2.2)	0.0631
N	26	27	1669	
Lactate dehydrogenase (U/L)	339.5 (373.2)	445.9 (448.3)	375.2 (307.7)	0.5311
N	22	19	1255	
Total protein (g/dL)	7.1 (0.6)	7.4 (0.6)	7.1 (0.6)	0.0403
N	25	25	1159	
INR (SD)	1.1 (0.3)	1.1 (0.1)	1.1 (0.2)	0.9978
N	21	16	764	
N-L ratio	4.1 (2.7)	4.6 (3.8)	4.5 (4.1)	0.8398
N	27	27	1673	
P-L ratio	238.6 (140.2)	253.9 (144.1)	234.7 (171.3)	0.8396
N	27	27	1673	

c)

Parameter	ECOG 0 (n=746)	ECOG 1 (n=956)	ECOG 2 (n=45)	p
Hematocrit (%)	36.3 (10.4)	34.6 (9.6)	NA	0.0005
N	32	35	0	
Hemoglobin (g/dL) (SD)	12.5 (1.4)	12.1 (1.6)	11.7 (1.6)	<0.0001
N	732	956	42	
Lymphocytes (count*10^9) (SD)	1.4 (0.8)	1.3 (0.8)	1.4 (0.7)	0.0433
N	732	953	42	
Monocytes (count*10^9) (SD)	0.5 (0.2)	0.6 (0.4)	0.7 (0.3)	<0.0001
N	445	428	20	

Neutrophils (count*10^9) (SD)	4.4 (3.0)	5.3 (2.7)	6.9 (3.2)	<0.0001
N	732	955	42	
Platelets (count*10^9) (SD)	249.3 (90.9)	269.1 (100.5)	321.0 (182.1)	<0.0001
N	732	956	42	
White blood cells (count*10^9) (SD)	6.4 (2.1)	7.4 (3.0)	9.2 (3.5)	<0.0001
N	732	956	42	
Alpha-1 acid glycoprotein (mg/dL) (SD)	147.7 (89.0)	158.8 (88.1)	242.3 (141.3)	<0.0001
N	475	618	31	
Albumin (g/dL) (SD)	4.1 (0.4)	4.0 (0.5)	3.6 (0.4)	<0.0001
N	732	956	42	
Alkaline phosphatase (U/L) (SD)	102.4 (65.2)	109.7 (77.0)	137.3 (119.1)	0.0033
N	727	953	42	
Alkaline transaminase (U/L) (SD)	22.9 (14.4)	24.1 (17.3)	33.3 (29.5)	0.0041
N	732	956	42	
Aspartate transaminase (U/L) (SD)	24.8 (16.8)	26.3 (16.1)	32.1 (19.6)	0.0072
N	729	954	42	
Direct bilirrubin (mg/dL) (SD)	0.2 (0.1)	0.2 (0.5)	0.2 (0.1)	0.4825
N	293	396	20	
Total bilirubin (mg/dL) (SD)	0.5 (0.2)	0.4 (0.2)	0.5 (0.3)	0.0613
N	732	956	42	
Calcium (mmol/L) (SD)	2.4 (0.5)	2.4 (0.1)	2.3 (0.2)	0.0042
N	496	790	27	
Total cholesterol (mg/dL) (SD)	198.5 (41.7)	193.3 (46.6)	152.4 (43.8)	0.0519
N	231	341	6	
Creatinine kinase (U/L) (SD)	79.6 (51.6)	65.5 (42.9)	55.1 (31.1)	<0.0001
N	718	934	41	
Creatinine (mg/dL) (SD)	0.8 (0.2)	0.8 (0.2)	0.8 (0.3)	0.1461
N	732	956	42	
Creatinine clearance (mL/min) (SD)	92.0 (35.2)	90.6 (34.4)	92.3 (43.1)	0.5998
N	529	684	34	
C-reactive protein (mg/L) (SD)	25.3 (124.7)	27.1 (46.2)	27.3 (33.6)	0.7477
N	390	661	22	
Glucose (mmol/L)	5.9 (1.8)	6.3 (2.4)	6.5 (2.0)	<0.0001
N	728	952	42	
Lactate dehydrogenase (U/L)	341.7 (258.2)	389.6 (321.3)	571.9 (614.5)	0.0001
N	491	775	30	
Total protein (g/dL)	7.2 (0.5)	7.1 (0.6)	6.7 (0.7)	<0.0001
N	568	615	26	
INR (SD)	1.0 (0.2)	1.1 (0.2)	1.1 (0.1)	0.0439
N	334	453	14	
N-L ratio	3.6 (2.4)	5.1 (4.9)	6.2 (4.3)	<0.0001
N	732	953	42	
P-L ratio	206.6 (115.1)	253.3 (186.3)	318.5 (388.3)	<0.0001
N	732	953	42	

d)

Parameter	Breast (n=128)	Colon (n=22)	Endometrial (n=120)	Gastric (n=7)	Hepatic (n=16)	Lung (n=865)	Ovarian (n=499)	Pancreatic (n=66)	Sarcoma (n=70)	p
Hematocrit (%)	23.0 (19.8)	38.1 (3.8)	36.9 (7.0)	NA	33.3 (7.1)	42.6 (1.7)	NA	28.8 (16.2)	40.3 (2.7)	<0.0001
N	7	28	7	0	4	2	0	13	6	
Hemoglobin (g/dL) (SD)	11.9 (1.4)	12.7 (1.4)	11.9 (1.4)	10.7 (0.7)	11.6 (2.0)	12.6 (1.5)	12.0 (1.4)	12.2 (1.2)	12.2 (1.8)	<0.0001
N	126	21	119	7	16	810	499	65	67	
Lymphocytes (count*10^9) (SD)	1.2 (0.5)	1.5 (0.8)	1.4 (0.6)	1.8 (1.2)	1.2 (0.7)	1.3 (1.0)	1.5 (0.6)	1.6 (0.8)	1.1 (0.6)	<0.0001
N	126	20	119	7	16	808	499	65	67	
Monocytes (count*10^9) (SD)	0.5 (0.2)	0.5 (0.2)	0.5 (0.2)	0.6 (0.1)	0.7 (0.3)	0.6 (0.3)	0.5 (0.4)	0.8 (0.4)	0.6 (0.3)	0.0062
N	122	4	85	2	13	193	409	12	53	
Neutrophils (count*10^9) (SD)	4.0 (1.8)	5.1 (2.2)	4.7 (2.4)	5.8 (2.2)	4.9 (1.9)	5.3 (3.3)	4.6 (2.4)	5.3 (2.8)	4.5 (2.0)	<0.0001
N	126	21	119	7	16	809	499	65	67	
Platelets (count*10^9) (SD)	256.0 (106.6)	269.1 (126.9)	279.1 (98.6)	281.4 (98.4)	206.9 (82.9)	250.1 (85.4)	281.0 (111.3)	258.9 (113.6)	257.4 (124.2)	<0.0001
N	126	21	119	7	16	810	499	65	67	
White blood cells (count*10^9) (SD)	5.9 (2.1)	7.5 (2.1)	6.7 (2.6)	8.8 (2.6)	6.9 (2.6)	7.3 (2.8)	6.9 (2.8)	7.8 (3.2)	6.5 (2.4)	<0.0001
N	126	21	119	7	16	810	499	65	67	
Alpha-1 acid glycoprotein (mg/dL) (SD)	126.2 (52.3)	122.1 (70.4)	142.4 (69.2)	124.1 (66.5)	125.3 (38.8)	132.3 (51.8)	249.3 (128.5)	112.3 (51.5)	121.5 (58.2)	<0.0001
N	113	12	107	7	14	512	245	52	62	
Albumin (g/dL) (SD)	4.1 (0.4)	4.0 (0.4)	4.1 (0.4)	3.6 (0.7)	3.7 (0.5)	4.1 (0.4)	4.0 (0.5)	3.9 (0.4)	4.1 (0.4)	<0.0001
N	113	12	107	7	14	512	245	52	62	
Alkaline phosphatase (U/L) (SD)	124.7 (87.5)	137.8 (58.9)	106.1 (62.8)	169.7 (122.6)	164.5 (94.6)	98.4 (66.8)	104.0 (63.2)	195.2 (143.3)	93.7 (34.4)	<0.0001
N	125	21	119	7	16	805	498	64	67	

Alkaline transaminase (U/L) (SD)	30.1 (21.3)	20.0 (14.1)	19.7 (11.1)	22.1 (18.5)	28.8 (17.8)	25.1 (18.1)	21.0 (12.9)	30.2 (19.7)	19.3 (9.3)	<0.0001
N	126	21	119	7	16	810	499	65	67	
Aspartate transaminase (U/L) (SD)	35.0 (25.0)	30.3 (17.1)	22.3 (11.9)	34.7 (20.5)	34.3 (16.3)	25.6 (15.2)	23.9 (15.8)	31.5 (20.6)	21.2 (8.7)	<0.0001
N	126	21	119	7	16	805	499	65	67	
Direct bilirrubin (mg/dL) (SD)	0.2 (0.1)	0.2 (0.1)	0.2 (0.1)	0.1 (0.0)	0.4 (0.3)	0.2 (0.1)	0.2 (0.1)	0.4 (1.4)	0.2 (0.1)	0.012
N	21	19	47	2	8	330	205	49	28	
Total bilirubin (mg/dL) (SD)	0.5 (0.2)	0.5 (0.2)	0.4 (0.2)	0.5 (0.4)	0.6 (0.3)	0.5 (0.2)	0.4 (0.2)	0.6 (0.3)	0.5 (0.2)	<0.0001
N	126	21	119	7	16	810	499	65	67	
Calcium (mmol/L) (SD)	2.3 (0.1)	2.4 (0.1)	2.4 (0.1)	2.2 (0.1)	2.3 (0.1)	2.4 (0.4)	2.4 (0.1)	2.3 (0.1)	2.3 (0.1)	
N	126	21	119	7	16	799	93	65	67	
Total cholesterol (mg/dL) (SD)	195.6 (40.1)	194.3 (59.2)	204.8 (41.3)	145.4 (29.4)	135.3 (NA)	195.7 (48.6)	208.8 (41.6)	176.5 (36.4)	176.4 (42.9)	<0.0001
N	101	3	65	7	1	213	93	56	39	
Creatinine kinase (U/L) (SD)	79.5 (40.3)	86.5 (49.1)	68.1 (41.1)	63.3 (38.2)	57.1 (29.0)	71.4 (51.1)	69.2 (41.8)	62.8 (53.3)	81.8 (50.1)	0.5169
N	125	20	119	7	15	793	484	63	67	
Creatinine (mg/dL) (SD)	0.7 (0.2)	0.9 (0.2)	0.8 (0.2)	0.9 (0.3)	0.9 (0.2)	0.9 (0.3)	0.8 (0.2)	0.8 (0.2)	0.8 (0.2)	<0.0001
N	126	21	119	7	16	810	499	65	67	
Creatinine clearance (mL/min) (SD)	113.3 (34.5)	82.3 (24.7)	88.2 (33.9)	98.0 (58.2)	89.3 (4.7)	92.4 (34.3)	85.1 (33.5)	106.4 (29.8)	128.1 (40.1)	<0.0001
N	15	21	51	2	2	689	409	17	41	
C-reactive protein (mg/L) (SD)	2.8 (6.3)	41.0 (66.3)	31.6 (44.0)	32.2 (52.4)	7.0 (7.5)	29.6 (99.7)	25.7 (42.3)	19.5 (27.9)	25.5 (37.8)	0.2476
N	98	20	66	7	2	698	83	58	41	
Glucose (mmol/L)	5.8 (1.6)	6.3 (1.6)	6.3 (2.5)	5.5 (1.1)	6.1 (1.5)	6.3 (2.4)	5.7 (1.4)	7.6 (4.4)	5.9 (1.6)	<0.0001
N	126	21	119	7	16	805	496	65	67	
Lactate dehydrogenase (U/L)	486.5 (300.3)	403.0 (228.0)	354.1 (254.2)	648.7 (677.5)	374.5 (143.2)	373.6 (331.1)	326.9 (219.8)	283.3 (135.8)	343.9 (341.9)	0.0002

N	126	21	115	7	16	791	91	63	66	
Total protein (g/dL)	7.1 (0.6)	7.5 (0.6)	7.1 (0.6)	6.5 (0.3)	6.9 (0.5)	7.1 (0.6)	7.1 (0.6)	6.9 (0.6)	7.0 (0.5)	0.0022
N	126	21	118	7	16	293	496	65	67	
INR (SD)	1.0 (0.1)	1.3 (0.5)	1.0 (0.2)	1.1 (0.2)	1.1 (0.3)	1.1 (0.2)	1.1 (0.2)	1.1 (0.1)	1.1 (0.1)	0.0002
N	123	21	117	7	16	294	93	63	67	
N-L ratio	4.6 (7.1)	5.2 (4.4)	4.2 (2.9)	4.5 (2.7)	5.1 (3.3)	5.1 (4.5)	3.5 (2.3)	4.0 (2.9)	5.0 (3.5)	<0.0001
N	126	20	119	7	16	808	499	65	67	
P-L ratio	263.7 (203.6)	286.3 (279.7)	247.6 (142.9)	189.7 (88.5)	203.5 (81.8)	240.7 (177.1)	215.0 (155.1)	188.1 (113.6)	282.2 (175.3)	0.0015
N	126	20	119	7	16	808	499	65	67	

ECOG-PS: ECOG performance status score

Supplementary Table S2. Summary of the Weibull parameters for age distributions in four groups.

Group	Shape (α)	Scale (λ)
Males without sarcoma	8.56	66.27
Males with sarcoma and females with breast tumor	4.23	52.91
Females without breast and sarcoma	7.41	65.22
Females with sarcoma	2.78	47.07

Supplementary Table S3. Summary of model residuals.

PARAMETER	RESIDUAL (ε)
Height	6.610
Weight	15.540
AAG	0.394
Albumin	0.400
Hb	1.400
N-L ratio	0.600
P-L ratio	0.420
CRP	1.110
LDH	0.550
HCT	0.039

AAG: alpha-1 acid glycoprotein, Hb= hemoglobin, HTC= hematocrit, P-L: Platelets/Lymphocytes, N-L: Neutrophils/Lymphocytes, CPR: C-reactive protein, LDH: lactate dehydrogenase.

Example of R code to predict height with its linear equation amd accounting for ε:

```
predict(fithght,list(AGE=ds1_sim$AGE, SEX=ds1_sim$SEX,
RACEGR=ds1_sim$RACEGR))+rnorm(dim(ds1_sim)[1],0,sqrt(sum(fithght$residuals^2)/(
fithght$df.residual)))
```

This equation complements Table 2 in the main text. In this example, the expression predict(fithght, list(AGE=ds1_sim$AGE, SEX=ds1_sim$SEX, RACEGR=ds1_sim$RACEGR)) predicts height based on the fitted model fithght, using the variables AGE, SEX, and RACEGR from the dataset ds1_sim; researchers must adapt the R code to their datasets and variable names. The second part, rnorm(dim(ds1_sim)[1], 0, sqrt(sum(fithght$residuals^2) / (fithght$df.residual))), adds random error with a mean of 0 and a standard deviation equal to the residual standard error of the model, accounting for variability not explained by the model. The residuals (ε) listed in Supplementary Table S3 reflect this uncertainty and must be accounted for to run predictions.

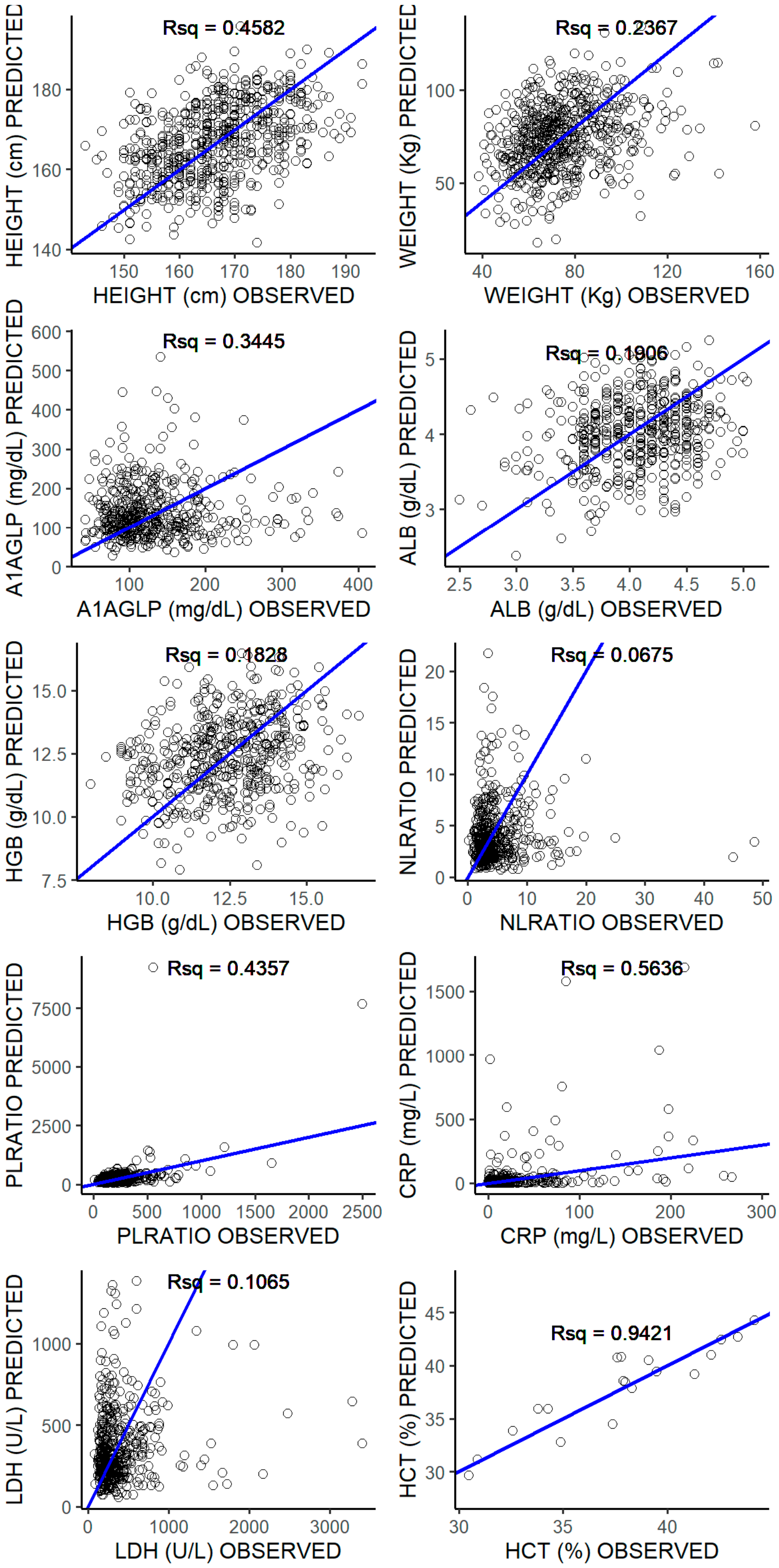

Supplementary Figure S1. Model verification goodness of fit plots.

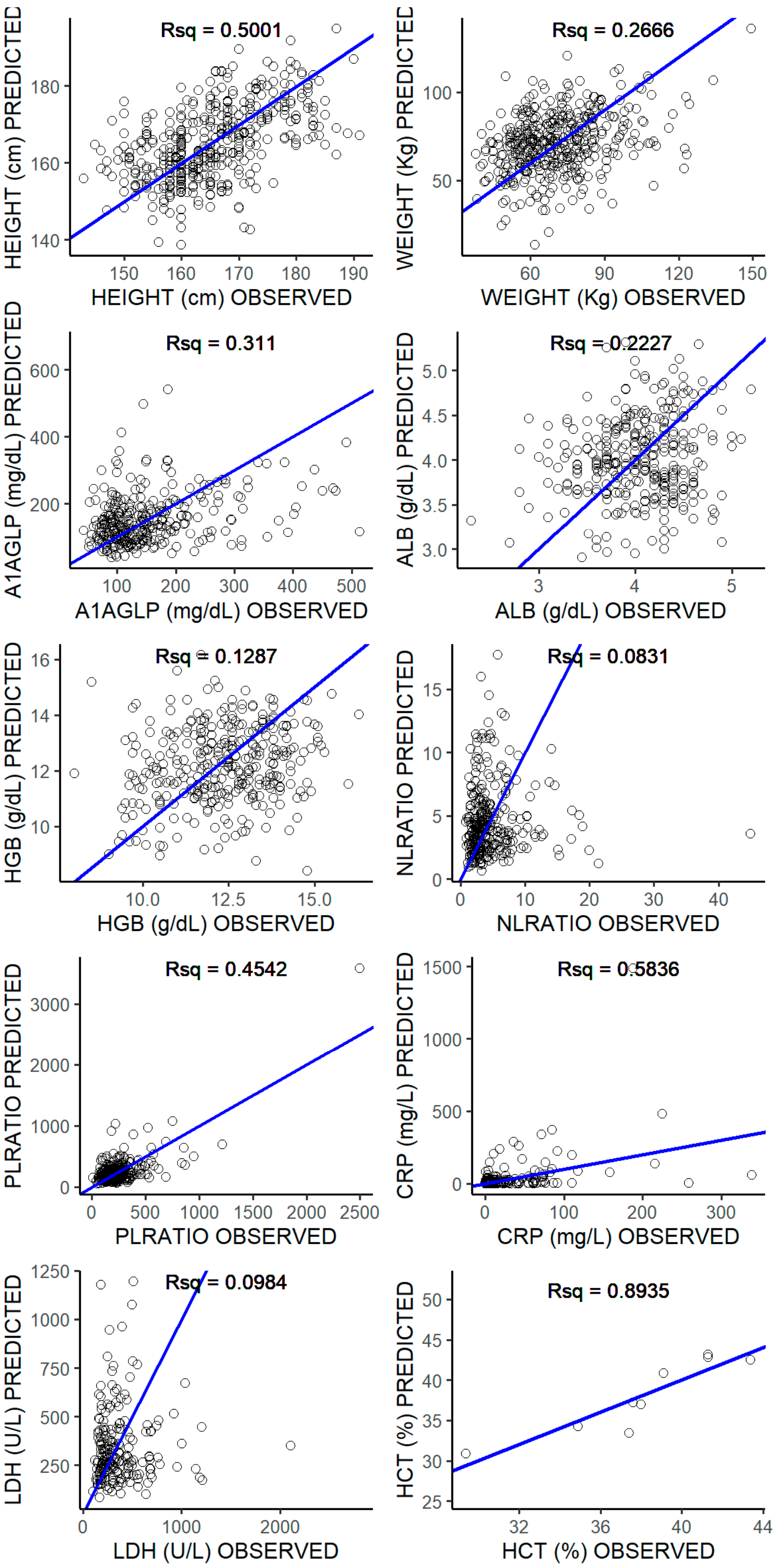

Supplementary Figure S2. Model validation goodness of fit plots.
